# Virtual approach of the aesthetical fit between hair colours and skin tones in women of different ethnical origin backgrounds

**DOI:** 10.1111/srt.13146

**Published:** 2022-03-08

**Authors:** Anthony Galliano, Myriam Guerin, Valerie Lambert, Ioanna Favrot, David Seneca, Fabien Lequeux, Frédéric Flament, Anna Sleurs, Berkly Foster, Edmund Phung, Kyung Moon Lee, Jeff Houghton

**Affiliations:** ^1^ L'Oréal Research and Innovation Centre Charles Zviak Saint‐Ouen France; ^2^ L'Oréal Paris Centre Eugene Schueller Clichy France; ^3^ ModiFace Inc Toronto Canada

**Keywords:** Artificial Intelligence, consumer preferences, hair color, Diversity of skin tones, Inclusivity

## Abstract

**Objective:**

To determine the aesthetical accordance between a given skin tone and the 11 possible colours of head hairs, covered by a marketed hair colouration product.

**Material and methods:**

The photographs of professional top models, representing several ancestries (non‐Hispanic European and Euro–American, East Asian, Hispanic Euro–American, and African–American ancestries), were used to virtually modify skin tones (from light, medium to dark) and hair colour by an artificial intelligence (AI)‐based algorithm. Hence, 117 modified photographs were then assessed by five local panels of about 60 women each (one in China, one in France and three in US). The same questionnaire was given to the panels, written in their own language, asking which and how both skin tones and hair colours fit preferentially (or not appreciated), asking in addition the reasons of their choices, using fixed wordings.

**Results:**

Answers from the five panels differed according to origin or cultural aspects, although some agreements were found among both non‐Hispanic European and Euro‐American groups. The Hispanic American panel in US globally much appreciated darker hair tones (HTs). Two panels (East Asian in China and African American in US) and part of non‐Hispanic European panel in France declared appreciating all HTs, almost irrespective with the skin tone (light, medium and dark). This surprising result is very likely caused by gradings (in %) that differ by too low values, making the establishment of a decisive or significant assessment. By nature highly subjective (culturally and/or fashion driven), the assessments should be more viewed as trends, an unavoidable limit of the present virtual approach. The latter offers nevertheless a full respect of ethical rules as such objective could hardly be conducted in vivo: applying 10 or 11 hair colourations on the same individual is an unthinkable option.

**Conclusion:**

The virtual approach developed in the present study that mixes two major facial coloured phenotypes seems at the crossroad of both genetic backgrounds and the secular desire of a modified appearance. Nonetheless, this methodology could afford, at the individual level in total confidentiality, a great help to subjects exposed to some facial skin disorders or afflictions.

## 1 INTRODUCTION

Despite their common genome, humans express a wide diversity of phenotypes that comprise different sizes, builds, weights, shapes and colour of hairs, face or eyes… In short, ‘all the same, all different’ (A. Langaney).

Two phenotypical elements are important drivers of the human appearance, skin and hair, particularly through a common denominator: their innate colour mainly conveyed by melanin pigments, synthetized by their own underlying melanocytes. Once mature, these pigments (eumelanin/black and phaeomelanin/red) are further injected as tiny granules either within epidermal cells or into the hair cortex. All these events and their consequences have been the objects of an abundant literature.^1^, ^2^, ^3^, ^4^, ^5^, ^6^, ^7^, ^8^, ^9^, ^10^, ^11^, ^12^, ^13^, ^14^

As for a healthy skin, these processes lead to a constitutive palette of tones, from pale (Keltic) to dark (African, Indian), due to an increased amount of both melanin pigments, noting that in all cases the ratio eumelanin/phaeomelanin remains constant (76% vs. 24%, respectively).^15^ Using spectrometers, the range of skin tones is now described by the continuous Individual Typology Angle parameter (ITA), calculated from the L*a*b* system (ITA°= arctan [L* − 50 / b*] * 180 / π), from −10° up to > +55° from very dark to pale complexions.^16^, ^17^ As skin tone is prone at changes with sun exposures (tanning), ITA allows to record either the intensity of a skin darkening, to evaluate the photo‐protective effect of sunscreens or to quantify a possible whitening effect.^18^, ^19^, ^20^, ^21^, ^22^, ^23^, ^24^, ^25^, ^26^, ^27^ Skin tone and its homogeneity may vary, too, with age (dark spots, i.e., lentigines) or with some discolouring afflictions such as vitiligo (whitening) or melasma (darkening). It is in addition a social/aesthetical element of high importance, especially in Asian countries, where a darker skin is negatively perceived, as a signature of ‘low‐class’ people (daily exposed to sun such as farmers, fishermen, outdoor workers etc.), realizing the high demand of topical whitening products in these countries. The most ancient procedure used to slightly correct the innate skin tone, and its possible heterogeneity is the technique of making‐up, based on powders of different shades applied by gentle brushes. Make‐up is nevertheless of a very provisory aspect and should be adapted to all skin tones, worldwide.^28^, ^29^ It allows to propose a first clusterization of skin tone, leading to 15 sub‐groups of bare skin colour to represent different ethnic groups. Another recent approach,^30^ combining colour science, consumer and colour artists insights allowed to co‐create the ideal shade range through a strategy analysis based on the identification of six global skin tone clusters with 18 sub‐clusters across the world. All these approaches lead to a dedicated and specific clusterization of skin tone but carried out on the same population diversity.

As for hair colour or hair tone (HT), the status appears somewhat different, with similarities and divergences. Unlike skin, hair colour tends to fade with sun or ultraviolet (UV) exposures via oxidative processes.^31^, ^32^, ^33^ Aging leads to a progressive loss of melanocytes of the hair follicle,^34^, ^35^ ultimately leading to hairs fully deprived of pigments, perceived as white hairs, a dreaded signature of aging. Of note, on a worldwide aspect, it comes clear that brown to dark hairs largely predominate, even in non‐Hispanic European and Euro‐American population who present the widest palette of hair colours, red hairs included.^36^, ^37^

But a striking divergence with skin emerged, about a century ago, by the development of oxidative hair dying using, as example, ParaPhenyleDiamine that, in the presence of other reactive entities (couplers) under oxidative conditions, leads to various hair colours.^32^ It is a safe procedure daily performed in hair salons by millions of women, worldwide. As for men, many aged men with brown/dark hairs resort to oxidative hair dying to cover their undesired white hairs.

In brief, the human faces, on a worldwide vision, present a large mosaic of variations created by different hair and skin colours, ranging from the darkest to the palest tones. Would an ‘ideal’ aesthetical fit between skin and hair colours be found (or not) across different cultural/ethnical criteria?, was the driving question of the present work.

Taking into account that skin tones not only vary between ethnical ancestries (e.g., African American vs. East Asian or non‐Hispanic European) but show differences within the same large ethnical group, we therefore aimed at evaluating by a virtual process, based on an artificial intelligence (AI)‐based algorithm, the aesthetical fit between some skin tones and some hair colours artificially modified by a marketed hair dyeing product. Such virtual approach was chosen as applying, in vivo, 11 different hair colouring on the models was unfeasible for both ethical and technical reasons. Same limitation obviously holds true with regard to skin innate colour. Hence, when virtually applied on seven professional top models representing four ethnical ancestries, changes in skin and hair tints were obtained:

1. Models 1 and 2: Two non‐Hispanic European models (*blond and brown hair for French panel*).
2. Models 3 and 4: Two non‐Hispanic Euro‐American models (*blond and brown hair for US panel*).
3. Model 5: One Hispanic Euro‐American model (*for US panel*).
4. Model 6: One African American model (*for US panel*).
5. Model 7: One East Asian model (*for Chinese panel*).

All individual resulting virtual images that comprise differently associated skin and HTs/colours were assessed by five panels of about 60 women each, aged 18–65 years, to establish the most appreciated fit between the respective tones of skin and hairs. The results of this study are the objects of the present paper.

## 2 MATERIAL AND METHODS

### 2.1 Subjects

The images of the top models illustrated here correspond to women under contract with our Group that covers the right to imaging. The virtual images were evaluated, eyes kept visible, in three local contract research organization (CRO) facilities by the selected panels (Eurosyn, FRANCE ‐ Paris; Reckner, USA ‐ New‐York / supervised by Eurosyn; Hycon Research, CHINA ‐ Shanghai / supervised by Eurosyn). The latter comprised 316 women, aged 18–65 years, with good visual acuity were enrolled to constitute a panel prone at expressing their own feelings on the aesthetical fit between various skin and HTs (see below). Each panel evaluates the corresponding model:

1. Panel 1: Phototype (I–III), non‐Hispanic European ancestries group – in France – evaluating Models 1 and 2
2. Panel 2: Phototype (I–III), non‐Hispanic Euro‐American ancestries group –in US – evaluating Models 3 and 4
3. Panel 3: Phototype (II–V), Hispanic Euro‐American ancestries group – in US – evaluating Model 5
4. Panel 4: Phototype (V–VI), African American ancestries group –in US – evaluating Model 6
5. Panel 5: Phototype (II–IV), East Asian ancestries group – in China – evaluating Model 7

### 2.2 Data basis

The present study initially re‐collected and used data on skin and HTs^19^, ^36^ previously obtained and published by our group of research. The first ones dealt with the natural skin tones of 507 women of ethnical backgrounds, objectively defined through the L*a*b* reference scale, using the Chromasphere methodology,^19^ thus allowing to create a skin colour chart.^19^ Similar to such objectives, the second study analyzed, through a spectrophotometer, the natural colour of 2870 hairs sampled from 2057 subjects (both genders) of 23 different regions of the five continents.^36^

In all cases, the L* (Luminance), a* (red‐green vector), and b* (yellow‐blue vector) values were used as such or as components of other colour markers such as hue (arctan b*/a*) or chroma (√(a*^2^ + b*^2^). The latter work allowed to better define 10 different HTs fitting well with the scale used by hair professionals dealing with hair dyeing, where HT1 represents the blackest tone, up to HT10, the palest one (extreme blond).

### 2.3 Protocol for cluster definition

**FIGURE 1:**
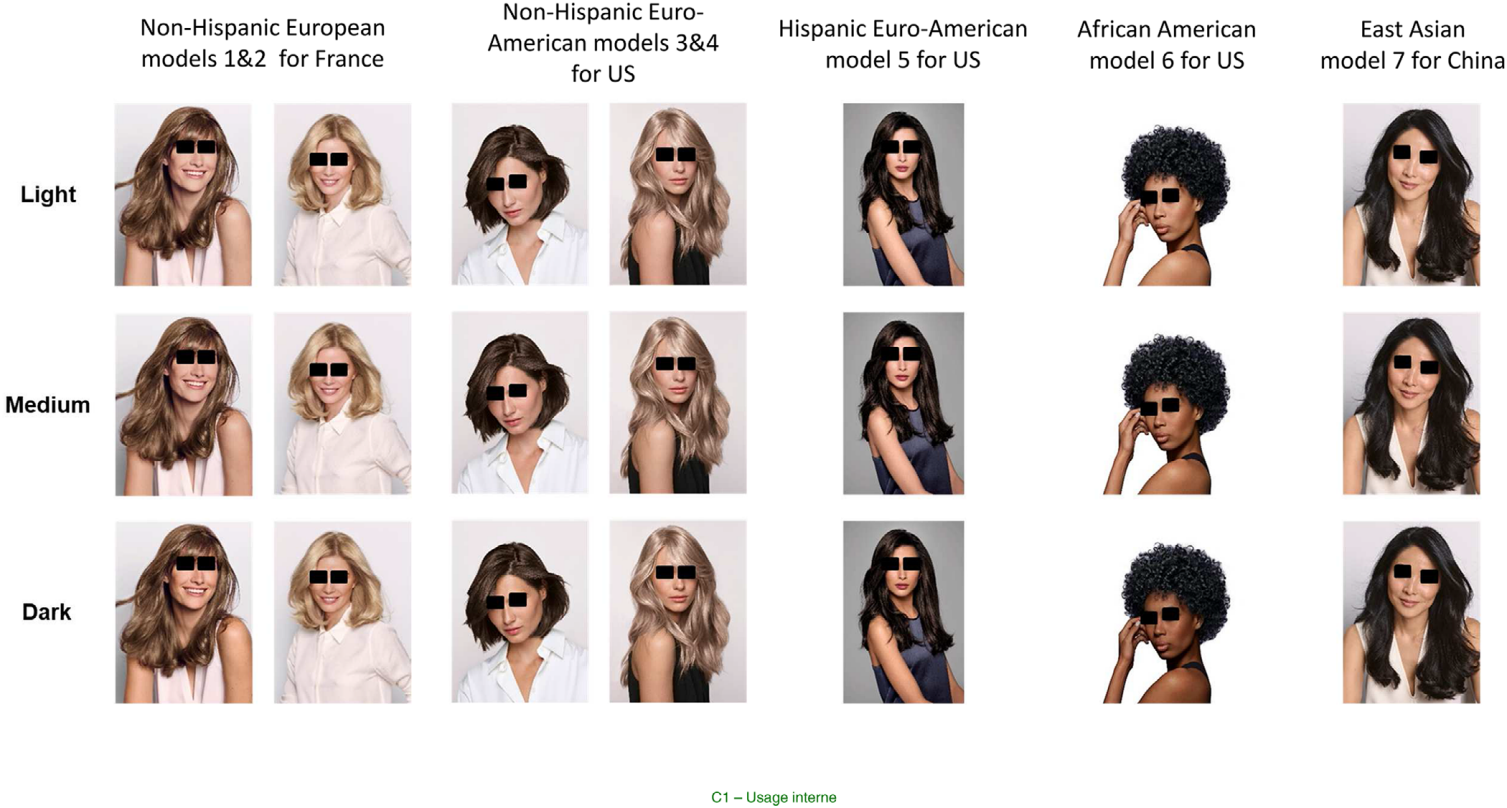
Illustrations of the 15 clusters with slightly different skin tones, that is, light, medium, and dark. From left to right: non‐Hispanic European and Euro‐American models 1‐2‐3‐4, Hispanic Euro–American model 5, African–American model 6 and East Asian model 7

**Skin clusterization**: L*a*b¨* references of skin tones were re‐extracted from previous data basis,^19^, ^28^ allowing to arbitrarily define some slight differences within each origin, named here as light (L), medium (M), and dark (D). These were selected, according to ethnic origin, through the differences in Luminance units (L*) between light and dark tones (L minus D). This difference varied between 5 and 10 L* units where 10 L* units were chosen in the darker skin tones (Afro‐American subjects) since showing larger variations along a full range scale (i.e., 25–50 L*units) than paler tones. Therefore, 15 sub‐clusters (5 × 3) were arbitrarily created for taking into account some subtle variations of skin tones within a same origin. These were then virtually applied on the images of top models (see below) using Photoshop software, taking reference values obtained by the Chromasphere instrument and the hair colour chart. These 15 virtual illustrations are shown in Figure 1.

**FIGURE 2:**
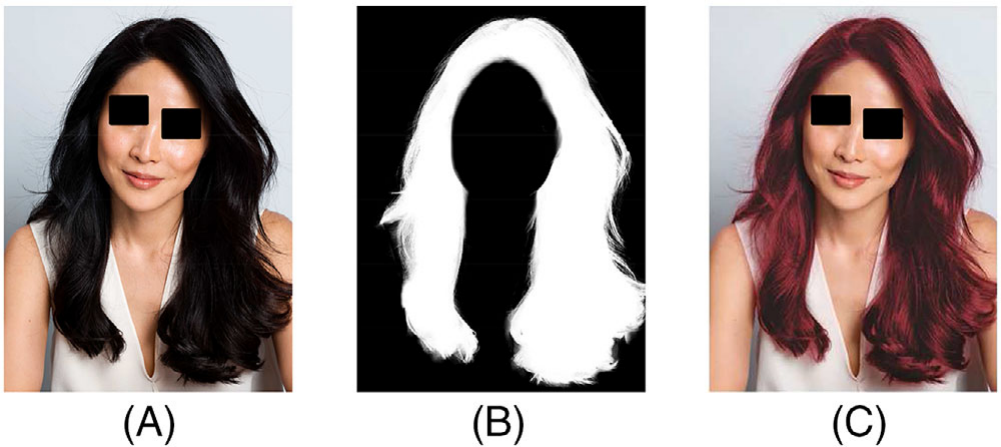
The successive steps used to modify hair shades on an East Asian model, where (A) represents her natural colour, (B) represents the AI‐based head hair segmentation and (C) the rendering obtained with another hair shade

**FIGURE 3:**
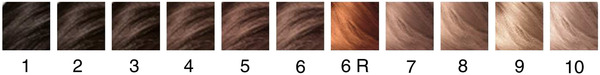
The 11 different hair tones possibly covered by the marketed hair‐dyeing product, from darkest to paler ones. 6R: red (Keltic‐like)

**Hairs colour representation**: Among the possible 11 HTs scale (HT1 to HT11), care was taken to choose hair shades of short distances, to avoid extreme contrast with skin tone (e.g., Asian or African subject with blond or pale hair). An AI‐based algorithm, derived from a previously developed and validated algorithm,^37^ allowed to detect, on each virtual image of the 15 top models with three different skin tones, the whole head hair to be replaced by the chosen HT as summarized by Figure 2, given as example:

The 11 HTs covered by the marketed hair dying product are illustrated by Figure 3. Hence, merging skin tones with HTs, a total of 117 virtual images were then created. To obtain natural visual results, from HT1 to HT6, models with dark hair background are used. From HT7 to 10, models with blond hair background are used (only for non‐Hispanic European and Euro‐American models).

**FIGURE 4:**
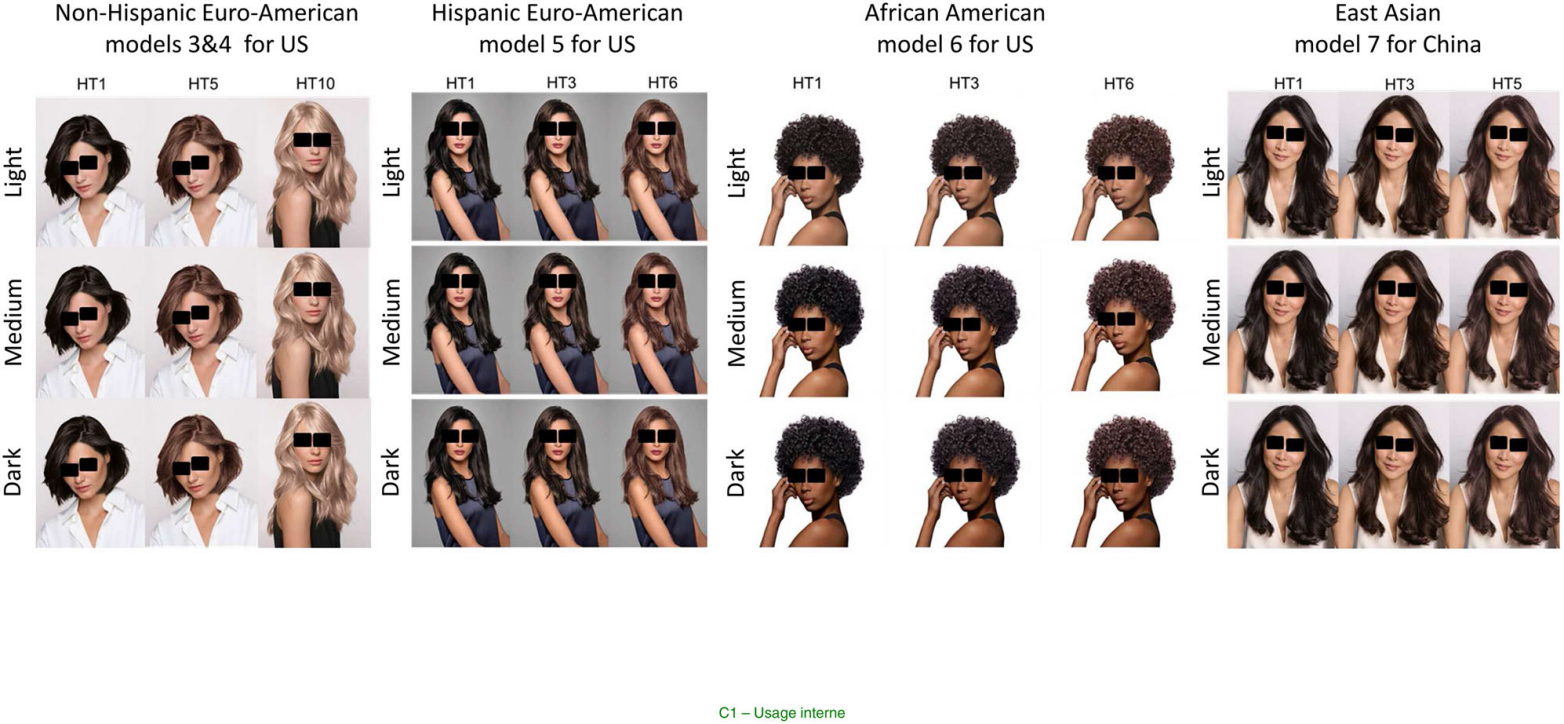
Examples of some virtual images obtained by merging different skin and hair tones in the five groups. From left to right: non‐Hispanic Euro–American models 3 and 4 L‐M‐DVs HT1‐5‐10, Hispanic Euro–American model 5 L‐M‐DVs HT1‐3‐6, African–American model 6 L‐M‐D Vs HT1‐3‐6 and East Asian model 7 L‐M‐DVs HT1‐3‐5

Some of them are illustrated by Figure 4, as representative examples.

### 2.4 Assessing panel

This panel comprised a total of 316 women from China (60 East Asian women phototype [II–IV]), US (66 African–American women phototype [V–VI], 61 Hispanic Euro‐American women phototype [II–V] and 66 non‐Hispanic Euro‐American phototype [I–III]) and France (63 non‐Hispanic European phototype [I–III]) aged 18–65 years (50% were 35–54 years old). All were regular users of hair colouration products, gathering a valuable mix of hair colours, with a good visual acuity. All women use a permanent hair colour (with or without ammonia), and they applied hair colouration by themselves only at home or occasionally at the hairdresser. They have used hair colour products from different brands at least every 4 months in the last 12 months. They were enrolled by our local CRO facilities, New York (US), Shanghai (China) and Paris (France). Their own skin tone and innate hair colour are coherent with the various ‘created’ skin tones/hair colours, as shown by Tables 1 and 2.

**TABLE 1:** Repartition of the various skin tones of the five panels

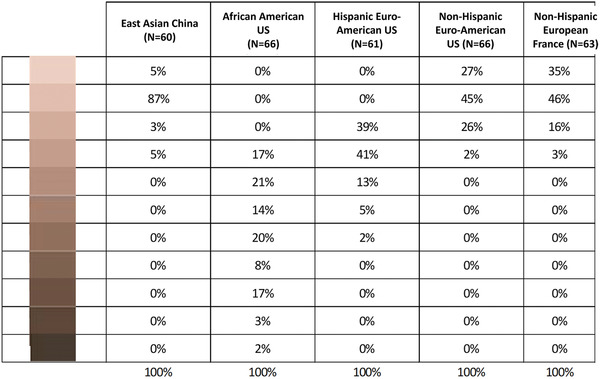

**TABLE 2:** Repartition of the constitutive hair colours of the assessing panels. Numbers indicate the average hair tone (HT) established by our hair dying experts during the enrollment phase

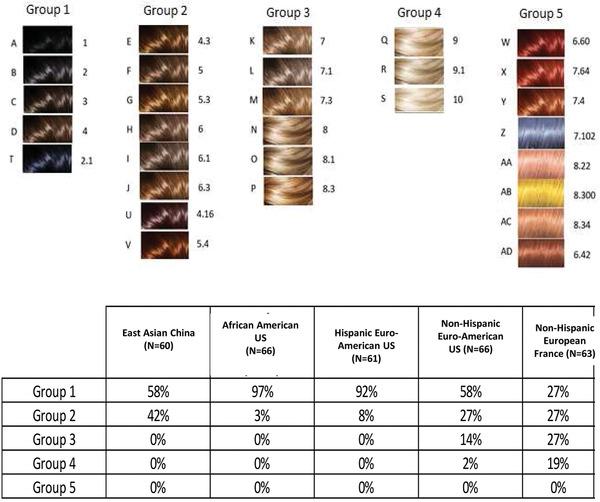

Two questionnaires, written in their own languages, were created in common by the three CRO's to obtain their appreciations in a two‐step procedure, by: (1) Step 1 ‐ evaluating the fit of the whole hair colour range with each skin tone and (2) Step 2 ‐ evaluating the fit of each hair colour shades for a given skin tone if the hair colour range is not satisfactory (sequential monadic, full randomization). Step 1 dealt with range assessment, where all pictures of each created skin tone of the same top model were displayed on the calibrated screen, whereas Step 2 dealt with colour assessment, where all pictures were presented one by one in a randomized order. The panel had to answer to the two types of questionnaires through 3 simple wordings, that is, agree, disagree, and do not know. The two series of questions are shown in Table 3.

**TABLE 3:** The two questionnaires (translated in four languages, English, Chinese, and French) used in the two‐step procedure of assessments

	Step 1 ‐ Whole hair colour range	Step 2 – Single picture presentation
Question 1	Do all these hair colours fit well with the skin tone of this woman?	Does the skin tone of this woman and her hair colour fit well together?
Question 2	Does this woman have a healthy glow, whatever the hair colour she wears?	Wearing this colour, does this woman has a healthy glow?
Question 3	Do all these hair colours flatter her skin tone?	Does the skin tone of this woman is flattered by her hair colour?
Question 4	Do all these hair colours look harmonious on this woman?	Do the hair colours look harmonious on this woman?

### 2.5 Statistics/Handling of data

At first, if 60% of a given panel who evaluated a picture corresponding to the association (hair colour‐ skin tone) rated an item positively, a relevance condition was considered. A 60% successful criterion therefore implies that, with a 95% confidence, the agreement rate would at least reaches 50% if the test had to be repeated with another panel of about 60 subjects presenting the same characteristics. According to the sample size and a given confidence level (here 95%), a percentage can vary from a minimum (down %) to a maximum (up %). With a panel size of 60 interviewed subjects, the figure of 60% falls in the confidence interval of 50%−70%. If the test was re‐performed with another comparable panel, there are 95% of chances that the obtained figure will be found in this interval, according to the following equation:

$$l\;=\;p+t_{1-\alpha }\sqrt{\frac{p\left(1-p\right)}{n}}$$

where *p* is the observed percentage of the sample, *n* its size, *t* a multiplier at a confidence level (here *α* = 95%), and I is the maximum of *p* within the confidence interval (= up percentage).

## 3 RESULTS

Table 4 summarizes the answers of the five local panels as positive agreements (‘agree,’ as %) on the fit between new hair colours and the three skin tones (light, medium, and dark) according to different aesthetical aspects obtained on the Step 1 (evaluation of the whole range of skin tone/hair colour). The positive ratings appear globally high (>70%), slightly higher in the relationship with a healthy glow (76% up to 97%), rather in line (close wordings) with the assessment of a harmonious look (question 4). Interestingly, both non‐Hispanic European and Euro–American cohorts present slightly lower positive answers than the three other groups.

**TABLE 4:** Summary of the positive assessments (‘agree’ as %) obtained by the five local panels on the aesthetical adequation between hair colours according three skin tones on Step 1 (whole hair colour range)

**Question 1: *Do all these hair colours fit well with the skin tone of this woman?***					
	**Non‐Hispanic** **European** **models 1 and 2** **rated in France**	**Non‐Hispanic** **Euro–American** **models 3 and 4** **rated in US**	**Hispanic Euro–American** **model 5 rated in US**	**African–American** **model 6 rated in US**	**East Asian model 7** **rated in China**
**Light**	78	71	77	97	90
**Medium**	76	65	75	94	85
**Dark**	71	68	69	86	87
**Question 2: *Does this woman have a healthy glow, whatever the hair colour she wears?***					
	**Non‐Hispanic** **European** **models 1 and 2** **rated in France**	**Non‐Hispanic** **Euro–American** **models 3 and 4** **rated in US**	**Hispanic Euro–American** **model 5 rated in US**	**African–American** **model 6** **rated in US**	**East Asian model 7** **rated in China**
**Light**	76	83	92	97	87
**Medium**	78	80	97	91	78
**Dark**	84	85	95	80	83
**Question 3: *Do all these hair colours flatter her skin tone?***					
	**Non‐Hispanic** **European** **models 1 and 2** **rated in France**	**Non‐Hispanic** **Euro–American** **models 3 and 4** **rated in US**	**Hispanic Euro–American** **model 5 rated in US**	**African–American** **model 6 rated in US**	**East Asian model 7** **rated in China**
**Light**	60	58	66	83	90
**Medium**	67	64	75	77	77
**Dark**	65	58	71	61	83
**Question 4: *Do* a*ll these hair colours look harmonious on this woman?***					
	**Non‐Hispanic** **European** **models 1 and 2** **rated in France**	**Non‐Hispanic** **Euro–American** **models 3 and 4** **rated in US**	**Hispanic Euro–American** **model 5 rated in US**	**African–American** **model 6 rated in US**	**East Asian model 7** **rated in China**
**Light**	79	77	79	83	93
**Medium**	79	74	80	80	82
**Dark**	71	76	77	76	88

In Step 2, the independent evaluation of each pictures permitted to go deeply in the specific analysis of the appreciation between skin tone and hair colour. Compiling all these answers with their respective modified HTs allows to precise the HTs that were given privilege by the five local panels, for the three skin tones and, inversely, to indicate which HTs were less appreciated (Table 5) taking a threshold of + or ‐10% differential assessment with other HTs. The term ‘All’ means that although all HTs were appreciated, their relative differences (+ or ‐3 %) were too small to allow a decisive ranking. Table 5 indicates that positive and negative appreciations much vary with ethnical/cultural backgrounds, even in the two non‐Hispanic European and Euro‐American cohorts. The case of 6R (Keltic‐like) colour is more favoured by the non‐Hispanic Euro‐American US group than its French counterpart, at least in light and medium skin tones. Interestingly, African American and Chinese panels show comparable trends, where darker skin tones seem less appreciated for paler HTs (4 or 5). The same holds true in the US Hispanic Euro‐American panel where, irrespective with skin tones, darker HTs (1–3) are given privilege while HT6 seems a true limit towards paler HTs.

**TABLE 5:** Repartition of modified hair tones (HTs) vis à vis their global appreciations by the local panels, according to origin and skin tones on Step 2 (evaluation on single picture)

	Light		Medium		Dark	
	More appreciated	Less appreciated	More appreciated	Less appreciated	More appreciated	Less appreciated
**Non‐Hispanic European France**	**All**	*Excepted* *HT 3 + 5 +8*	HT (8; 9)	*HT 10*	**All**	*Excepted* *HT 6 + 8*
**Non‐Hispanic Euro–American US**	HT (2; 4) + 9 + 6R	*HT 6*	HT (1; 3) + (7; 8) + 6R	*HT (5; 6) + 9*	HT (1; 3) + (6; 7)	*HT 4 + (9; 10) + 6R*
**Hispanic Euro–American US**	HT (1; 3)	*HT 6*	HT (1; 3)	*HT 6*	HT (1; 3)	*HT 6*
**African–American US**	**All**		**All**		**All**	*Excepted* *HT (5; 6)*
**East Asian China**	**All**	*Excepted* *HT 4*	**All**		HT (1;2)	*HT (4; 5)*

When the hair colour/skin tone range received a favourable score during the Step 1 evaluation, judges answered to five additional questions about subjective criteria (young, healthiness, natural glamour, glowing look). The Table 6 summarizes the positive answers obtained by the five local panels (‘agree’, as %) on the impacts of the new hair colours on five subjective criteria of the facial appearance of the virtual models. In many cases, low values were obtained, and only those corresponding to a clear agreement were retained. In fact, if most agree (about 70%), a large proportion of these responders declined or were unable to clearly associate their positive answers with the wordings used (young, healthy etc.). Despite these lower values than those expressed in Table 4, it is noteworthy that, in all five cohorts, the skin tone presents a low impact on these answers, with rather comparable averaged values. In the five cohorts, the terms ‘Healthy’ and ‘Glowing’ seem more privileged, with higher positive ratings (>40%, up to 64% among the French panel). The three other criteria (‘young’, ‘natural’ and ‘glamour’), of a probably too close subjective interpretation, present similarly lower ratings (about 30%), irrespective with the origin.

**TABLE 6:** Summary of the positive answers (‘agree’, as %) obtained by the five local panels on various subjective social aspects of the facial appearance of virtual models of the three skin tones, where hair colours were artificially modified (obtain on Step 1, on the whole range of hair colour)

Would you say that, wearing this hair colour, this woman looks…					
**Young**	**East Asian China**	**African–American US**	**Hispanic Euro–American US**	**Non‐Hispanic Euro–American US**	**Non‐Hispanic European France**
**Light**	22%	27%	34%	35%	22%
**Medium**	19%	26%	33%	34%	25%
**Dark**	21%	22%	35%	35%	23%
**Healthy**	**East Asian China**	**African–American US**	**Hispanic Euro–American US**	**Non‐Hispanic Euro–American US**	**Non‐Hispanic European France**
**Light**	42%	41%	38%	37%	49%
**Medium**	45%	40%	38%	37%	48%
**Dark**	41%	41%	36%	38%	47%
**Natural**	**East Asian China**	**African–American US**	**Hispanic Euro–American US**	**Non‐Hispanic Euro–American US**	**Non‐Hispanic European France**
**Light**	36%	32%	28%	27%	28%
**Medium**	36%	35%	29%	29%	28%
**Dark**	38%	37%	32%	26%	30%
**Glamour**	**East Asian China**	**African–American US**	**Hispanic Euro–American US**	**Non‐Hispanic Euro–American US**	**Non‐Hispanic European France**
**Light**	25%	26%	26%	37%	36%
**Medium**	31%	26%	26%	32%	30%
**Dark**	27%	28%	27%	37%	29%
**Glowing**	**East Asian China**	**African–American US**	**Hispanic Euro–American US**	**Non‐Hispanic Euro–American US**	**Non‐Hispanic European France**
**Light**	30%	32%	32%	35%	64%
**Medium**	32%	33%	34%	33%	57%
**Dark**	30%	30%	35%	36%	59%

## 4 DISCUSSION

The human face presents, worldwide, a mosaic of variations in the intrinsic colours of skin and hairs, keeping in mind that innate darker skin complexions and darker hairs largely predominate. Whatsoever, in their constitutive status, skin and hair colours were shown potent drivers of social or psychosocial relationships,^38^, ^39^, ^40^, ^41^ although these quoted works were performed on women of same ethnical ancestry or on lightly pigmented young women.

As hair dying became, since decades, a common practice in many women, the paradigm of these quoted works may have changed. Such a question mostly led us to undertake the present study based on virtual images that included a mix of skin tones and artificially modified hair colours in women from five ethnic ancestries. Such virtual approach was chosen for both basic and ethical reasons: (1) how and to which extent a new hair colour may aesthetically fit with slightly different innate skin tones and (2) undertaking in vivo the same approach would have required the unethical successive applications of various hair shades on our selected models.

In brief, we believed that the virtual modifications of hair colour and skin tones would largely overcome such issues. Of note, the present work only dealt with aesthetical assessments and associated subjective terms (young, healthy, glowing…) and not on their possible social or psychosocial consequences that were only explored on innate skin and HTs.^38^, ^39^, ^40^, ^41^ The results shown in Table 5 indicate subtle divergences of positive and negative agreements according to origin and probably at a larger extent, to cultural aspects and their possibly associated fashions. In that sense, it may not be surprising to observe that the French panel agreed on the 6R colour as a remembrance of Keltic traits (Irish, Scottish) at least in L and M skin tones. Same holds likely true with regard to the Hispanic panel that gives privilege to darker HTs (1–3) as the predominant dark hairs of Spanish women^36^ are viewed by many as a true Spanish ‘signature’, including the past crossbreeding's in South America some centuries ago. The Chinese and African–American panels gave blurring answers as ‘all’ HTs seemed accepted, at the exception of the Chinese panel with dark skin tone clearly favours dark HTs (1 and 2). Both cohorts with dark skin tone clearly less appreciated paler HTs (4–6). Globally, the divergences between the assessments by the non‐Hispanic European US and French panels remain rather coherent despite a few discrepancies, again likely linked to current cultural and fashion criteria noting that the skin tone (L, M, D) has nevertheless a rather minor impact upon the assessments of HTs. We acknowledge that the present work obviously possesses limits with regard to the statistical aspect of the answers from all panels who classify many HTs with probably too much indulgence, leading to many differences of low amplitudes between HTs. Hence, these answers should be more regarded as trends, keeping in mind that these panels may not fully reflect the assessments of their whole population. In addition some terms, such as glow, used by laymen are probably too subjective, leaving room to uncertainties. Whatsoever, the methodological approach by a virtual process has proven interest by using aesthetical models on which two major phenotypes (skin and hair colours) can be infinitely modified. In addition, such approach may allow to confidentially help subjects with skin problems or afflictions (melasma, albinism, vitiligo, lentigines, freckles, and telangiectasia etc.) to help them choosing the more appropriate hair colouring tone. This method appears as a good tool to create material for test when it is not possible to assess the desired rendering. However, the final rendering is always based, and finetuned on real tests carried out on models, to make sure that the simulation globally fits with reality. Mixing skin tone and hair colour on the same person allows to propose a standard experience of evaluation to consumer in limiting the influence of facial differences that could induce preferences not only linked to the association between hair and skin colours. Using different representative ethnical origin models, we planned a worldwide study that may allow to show the diversity of consumer preferences, linked to cultural aspects and trends in each country. Even if this study is today conducted in CRO, to control the picture visualization on a calibrated screen, future studies will be completely performed in a full digital way, that is, through the nomad self‐acquisition (selfie), characterizing skin and hair features by gathering a AI‐based algorithm^42^ and a nomad consumer study. Despite some inherent limits, the present work appears at the crossroad between genetic backgrounds and the secular and/or cultural desire to improve and amend their inherited entailments at an intimate aesthetical level.

## CONFLICT OF INTEREST

All authors are the employees of the L'Oréal Group and its affiliate Modiface company.

## References

[1] Jablonski NG , Chaplin G . The evolution of human skin coloration. J Hum Evol. 2000;39:57–106. 10896812 10.1006/jhev.2000.0403

[2] Jablonski NG . The evolution of human skin and skin color. Ann Rev Anthropol. 2004;33:585–623.

[3] Jablonski NG . Why humans come in colors. Anthro Notes. 2011;32:7–10.

[4] Jablonski NG , Chaplin G . Epidermal pigmentation in the human lineage is an adaptation to ultraviolet radiation. J Hum Evol. 2013;65(5):671–5. 24112698 10.1016/j.jhevol.2013.06.004

[5] Chaplin G , Jablonski NG . Vitamin D and the evolution of human pigmentation. Am J Phys Anthropol. 2009;139(4):451–61. 19425101 10.1002/ajpa.21079

[6] Lucock M , Yates Z , Martin C , Choi J‐H , Boyd L , Tang S , et al. Vitamin D, folate and potential early lifecycle environmental adult phenotypes. Evol Med Public Health. 2014;1:69–91. 10.1093/emph/eou013 PMC4001294 24699387

[7] Wheat CM , Wesley NO , Jackson BA . Recognition of skin cancer and sun protective behaviors in skin of color. J Drugs Dermatol. 2013;12(9):1029–32. 24002151

[8] Agbai ON , Buster K , Sanchez M , Hernandez C , Kundu RV , Chiu M , et al. Skin cancer and photoprotection in people of color: a review and recommendations for physicians and the public. J Am Acad Dermatol. 2014;70(4):748–62. 24485530 10.1016/j.jaad.2013.11.038

[9] Battie C , Gohara M , Verschoore M , Roberts W . Skin cancer in skin of color: an update on current facts, trends and misconceptions. J Drugs Dermatol. 2013;12:194–8. 23377393

[10] Perez Oliva AB , Fernendez LP , Detorre C , Herráiz C , Martínez‐Escribano JA , Benítez J , et al. Identification and functional analysis of novel variants of the human melanocortin 1 receptor found in melanoma patients. Hum Mutat. 2009;30(5):811–22. 19338054 10.1002/humu.20971

[11] Basu Mallick C , Iliescu FM , Möls M , Hill S , Tamang R , Chaubey G , et al. The light skin allele of SCL24A5 in South Asians and Europeans shares identity by descent. PLoS Genet. 2013;9(11):e1003912. 24244186 10.1371/journal.pgen.1003912 PMC3820762

[12] Wilde S , Timpson A , Kirsanow K , Kaiser E , Kayser M , Unterländer M , et al. Direct evidence for positive selection of skin, hair and eye pigmentation in Europeans during the last 5,000 years. Proc Natl Acad Sci U S A. 2014;111(13):4832–7. 24616518 10.1073/pnas.1316513111 PMC3977302

[13] Chardon A , Cretois I , Hourseau C . Skin color typology and suntanning pathways. Int J Cosm Sci. 1991;13(4):191–208. 10.1111/j.1467-2494.1991.tb00561.x 19291061

[14] Yamaguchi Y , Hearing VJ . Physiological factors that regulate skin pigmentation. Biofactors 2009;35(2):193–9. 19449448 10.1002/biof.29 PMC2793097

[15] Del Bino S , Shosuke I , Sok J , Nakanishi Y , Bastien P , Wakamatsu K , et al. Chemical analysis of constitutive pigmentation of human epidermis reveals constant eumelanin to pheomelanin ratio. Pigment Cell Melanoma Res. 2015; 28(6):707–17. 26285058 10.1111/pcmr.12410

[16] Ohta N , Robertson AR . Colorimetry, fundamentals and applications. Chichester, West Sussex, UK: J Wileys & Sons Ltd; 2005. p. 1–329.

[17] Malacara D . Color vision and colorimetry: theory and application. 2nd ed. Bellington, WA : SPIE Press Book; 2011, 1–188.

[18] Wei L , Xuemin W , Wei L , Li L , Ping Z , Yanyu W , et al. Skin color measurement in Chinese female population: analysis of 407 cases from 4 major cities in China. Int J Dermatol. 2007;46(8):835–9. 17651167 10.1111/j.1365-4632.2007.03192.x

[19] De Rigal J , Abella M , Giron F , Caisey L , Lefebvre MA .Development and validation of a new Skin Color Char. Skin Res Technol. 2007;13:101–9. 17250540 10.1111/j.1600-0846.2007.00223.x

[20] Macdonald HM , Mavroeidi A , Aucott LA , Diffey BL , Fraser WD , Ormerod AD , et al. Skin colour change in Caucasian postmenopausal women predicts summer‐winter change in 25‐hydroxyvitamin D: findings from the ANSAViD cohort study. J Clin Endocrinol Metab. 2011;96(6):1677–86. 21411556 10.1210/jc.2010-2032

[21] Huixia Q , Xiaohui L , Chengda Y , Yanlu Z , Senee J , Laurent A , et al. Instrumental and clinical studies of the facial skin tone and pigmentation of Shanghaiese women. Changes induced by age and a cosmetic whitening product. Int J Cosmet Sci. 2012;34(1):49–54. 21848763 10.1111/j.1468-2494.2011.00680.x

[22] Guideline for the colorimetric determination of skin colour typing and prediction of the minimal erythemal dose (MED) without UV exposure / f[COLIPA. European Cosmetic, Toiletry and Perfumery Association] 2007.

[23] Fitzpatrick TB . The validity and practicability of sun‐reactive skin type I through VI. Arch Dermatol. 1988;124:869–71. 3377516 10.1001/archderm.124.6.869

[24] Del Bino S , Bernerd F . Variations in skin colour and the biological consequences of ultraviolet radiation exposure. Br J Dermatol. 2013;169(3):33–40. 24098899 10.1111/bjd.12529

[25] Del Bino S , Sok J , Bessac E , Bernerd F . Relationship between skin exposure to ultraviolet and the skin color type. Pigment Cell Res. 2006;19(6):606–14. 17083487 10.1111/j.1600-0749.2006.00338.x

[26] Qiu H , Flament F , Long X , Wu J , Xu M , Leger DS , et al. Seasonal skin darkening in Chinese women: the Shanghaiese experience of daily sun protection. Clin Cosm Investig Dermatol. 2013;6:151–8. 10.2147/CCID.S41578 PMC3674750 23754871

[27] Galzote C , Estanislao R , Suero MO , Khaiat A , Mangubat MI , Moideen R ,et al. Characterization of facial skin of various Asian populations through visual and non‐invasive instrumental evaluations: influence of age and skin care habits. Skin Res Technol. 2013;19(4):454‐65. 23521621 10.1111/srt.12069

[28] Caisey L , Grangeat F , Lemasson A . Skin color and make up strategies of women from different ethnic groups. Int J Cosmet Sci. 2006;28:427–37. 18489287 10.1111/j.1467-2494.2006.00329.x

[29] Baras D , Caisey L . Skin and lip technology. In: Kelly AP , Taylor S, editors. Dermatology for skin of color. Berkshire, UK: McGraw Hill professional; 2009. p. 541–51.

[30] Qiao Y , Atis B , Cointereau‐Chardon S . et al. Developing the most inclusive and relevant liquid foundation ranges for multicultural consumers, IFSCC Congress Yokohama, 2020.

[31] Corbett JF . Chemistry of hair colorant processes. Science as an aid to formulation and development. J Soc Cosmet Chem. 1984;35:297–310.

[32] Zviak C , Milléquant J . Hair colouring. In: Bouillon C, Wilkinson J , editors. The science of hair care. 2nd ed. Boca Raton: Taylor and Francis Group; 2005.

[33] Ito S , Wakamatsu K . Diversity of human hair pigmentation as studied by chemical analysis of eumelanin and pheomelanin. J Eur Acad Dermatol Venereol. 2011;25(12):1369–80. 22077870 10.1111/j.1468-3083.2011.04278.x

[34] Commo S , Bernard BA . Melanocyte subpopulation turnover during the human hair cycle: an immunohistochemical study. Pigment cell. 2000;13(4):253–9. 10.1034/j.1600-0749.2000.130407.x 10952393

[35] Commo S , Gaillard O , Bernard BA . Human hair greying is linked to a specific depletion of hair follicle melanocytes affecting both the bulb and the outer root sheath. Br J Dermatol. 2004;150(3):435–43. 15030325 10.1046/j.1365-2133.2004.05787.x

[36] Lozano I , Saunier JB , Panhard S , Loussouarn G . The diversity of the human hair colour assessed by visual scales and instrumental measurements. A worldwide survey. Int J Cosmet Sci. 2017;39(1):101–7. 27506896 10.1111/ics.12359

[37] Flament F , Zhang Y , Yu Z . Developing an artificial intelligence (A.I)‐based descriptor of facial appearance that fits with the assessments of makeup experts. Skin Res Technol. 2021. 10.1111/srt13061. 33998717

[38] Fink B , Liebner K , Müller AK , Hirn T , Mckelvey G , Lankhof J . Hair and skin color together influence perceptions of age, health and attractiveness in lightly‐pigmented young women. In J Cosmet Sci. 2018; 40:303–12. 10.1111/ics.12467. 29772598

[39] Swami V , Furnham A , Joshi K . The influence of skin tone, hair length and hair color on rating of women's physical attractiveness, health and fertility. Scand J Psychol. 2008;49(5):429–37. 18452501 10.1111/j.1467-9450.2008.00651.x

[40] Samson N , Fink B , Matts PJ . Visible skin condition and perception of human facial appearance. Int J Cosmet Sci. 2010;23(3):167–84. 10.1111/j.1468-2494.2009.00535.x 19889046

[41] Rowland H , Burries RP . Human skin colour in mate choice and competition. Philos Trans R Soc Lond B Biol Sci. 2017;20160350. 10.1098/rstb. 28533465 PMC5444069

[42] Flament F , Maudet M , Ye C , Zhang Y , Jiang R , Dubosc S , et al. Comparing the self‐perceived effects of a facial anti‐aging product to those automatically detected from selfie images of Chinese women of different ages and cities. Skin Res Technol. 2021;27(6):880–90. 33822402 10.1111/srt.13037

